# The Brain as a Black Body in a Black Box

**DOI:** 10.21315/mjms-12-2024-970

**Published:** 2025-04-30

**Authors:** Zamzuri Idris, Jafri Malin Abdullah, Abdul Rahman Izaini Ghani, Zaitun Zakaria, Ang Song Yee, Diana Noma Fitzrol, Muhammad Ihfaz Ismail, Sanihah Abdul Halim

**Affiliations:** 1 Department of Neurosciences, School of Medical Sciences, Universiti Sains Malaysia, Health Campus, Kelantan, Malaysia; 2 Brain and Behaviour Cluster, School of Medical Sciences, Universiti Sains Malaysia, Health Campus, Kelantan, Malaysia; 3 Hospital Pakar Universiti Sains Malaysia, Universiti Sains Malaysia, Health Campus, Kelantan, Malaysia

**Keywords:** black-body, black box, plasma physics, buoyancy, microgravity, brain, thermodynamics, exclusion zone water, glymphatic system

## Abstract

The cranium houses the brain, and layers formed by the scalp, cranial bones, and meninges protect it from external energy, including light. Therefore, the brain can be viewed as a black body in a black box. The layers also protect the brain from external atmospheric or gravity force, and together with cerebrospinal fluid (CSF), it creates a microgravity or buoyant brain compartment. The brain curving (microgravity posture) mainly occurs at the mesencephalic-diencephalic junction and is thought to be because of the microgravity environment, thus, creating a nearly spherical-like brain geometry. The roughly spherical brain geometry, a black body with its heat irradiation, the presence of charged fluid in the brain extracellular space, and brain requirements to optimally cleanse waste products and be protective against micro-organisms may suggest a possible presence of plasma-like brain energy. This energy has the capability to emit infrared, and its amount is determined by black-body radiation and thermodynamics principles.

## The Brain and Cerebrospinal Fluid in a Cranium

**Figure 1:**
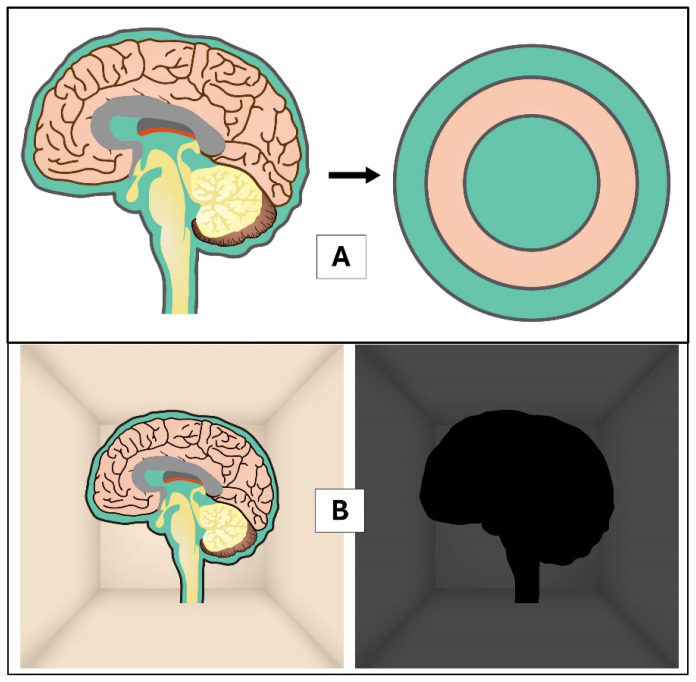
Cranium, brain and CSF buoyancy: (A) a sandwiched and buoyant brain; (B) a brain as a black body in a black box Notes: **A** = The CSF inside the ventricles, basal and surface cisterns form sandwich-like CSF spaces for brain parenchyma. Therefore, there are two major brain-CSF flows: a) via ventricles, ventricular foramina to cisterns, and b) via ventricles (trans-ependymal flow) or subarachnoid spaces into brain parenchyma (glymphatic system). Any cisternal haemorrhage or arachnoiditis may alter CSF-parenchymal flow and alter brain function by causing accumulated waste products or heat. **B** = The brain as a black body in a black box: since the thickest skull bones (petrous part of temporal bone, posterior part of sphenoid and clival part of occipital bones) are mainly in the posterior fossa (where brainstem-thalamus-hypothalamus/consciousness control areas are mainly resided), we thought that it is the darkest area of the box

The cranium is made of bones that surround the brain and meningeal layers. It gives physical protection from external forces and energy such as light, to the brain. In addition, the cranium, together with the cerebrospinal fluid (CSF), provides an enclosed space for a buoyant or microgravity environment. With this special intracranial environment, normal brain-bending (microgravity posture) appears at the mesencephalic-diencephalic junction during embryological development (1). Besides creating buoyancy, CSF is also responsible for controlling brain temperature. It provides a cooling system that follows thermodynamic principles for the brain (2). The CSF inside the ventricles, basal, and surface cisterns form sandwich-like CSF spaces for the brain parenchyma [Figure 1(A)], which also allows the optimal flow of CSF into the brain parenchyma via the glymphatic system, thus, good in cooling the brain and in clearing the brain waste products (3). The brain is the only organ with CSF that helps with cleansing; other organs mostly depend on their lymphatic-vascular system. Apart from CSF, the cranial diploic-emissary veins, intracranial vessels, basal air sinuses, and skull are also crucial in regulating brain temperature. They modulate brain temperature via conduction, convection, and radiation. As stated before, the cranium that houses the brain protects from external light and limits brain radiation externally. With this feature, the brain could be viewed as a black body in a black box [Figure 1(B)].

## The Brain as a Black Body in a Black Box

**Figure 2:**
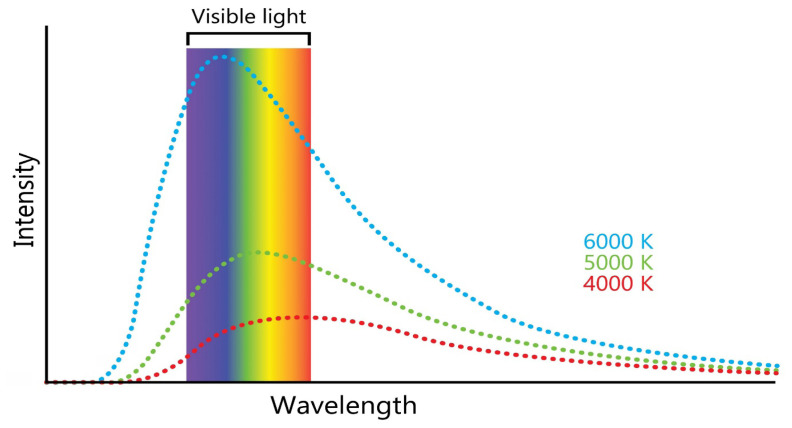
Black-body radiation curves and Max Planck’s law Note: This law describes the spectrum of black-body radiation in which, at higher temperatures, the total radiated energy increases, and the intensity peak of the emitted spectrum shifts to shorter wavelengths (to the left)

The physics of thermodynamics has highlighted that all normal matter at temperatures above absolute zero (0 Kelvin or −273.15°C) emits electromagnetic radiation, which represents a conversion of a body’s internal thermal energy into electromagnetic energy and is often called thermal radiation. Conversely, all normal matter absorbs electromagnetic radiation to some degree. A black body is an object that absorbs all incident electromagnetic radiation, regardless of frequency or angle of incidence, and its absorptivity is equal to unity, which is also the highest possible value. Therefore, a black body is a perfect absorber and emitter (4). When a black body is at a uniform temperature (at thermodynamic equilibrium), its emission has a characteristic frequency distribution that depends on the temperature. This emission is called black-body radiation. Max Planck’s law is one of the laws of physics associated with a black body (5). This law describes the spectrum of black body radiation, which depends only on the object’s temperature and has the following important features (Figure 2):

- at higher temperatures, the total radiated energy increases, and the intensity peak of the emitted spectrum shifts to shorter wavelengths (to the left);
- at any wavelength, the magnitude of the emitted radiation increases with increasing temperature; and
- the shape of the black-body radiation curve is suggestive of light as particles (photons) with quanta of energy (besides viewing light as waves).

In relation to a black-body object and its radiation, the nearly spherical geometric brain in the cranium shares two main similarities:

- the brain is in a black box, so it may appear dark or black in appearance (the darkest area is in posterior fossa which is covered by the thickest cranial bones); and
- the brain has its temperature range and regulated tightly by both:
  - biological processes such as amount of blood flow (heat regulation via conduction), airflow at the skull base (heat regulation via convection), optimal function of glymphatic system and optimal flow of CSF (heat regulation via conduction and convection); and
  - physics system, the skull and inner glistening dura which limits the irradiated energy (the skull bones) and helps in reabsorption of irradiated energy (glistening inner dura) (heat regulation via radiation).

**Figure 3:**
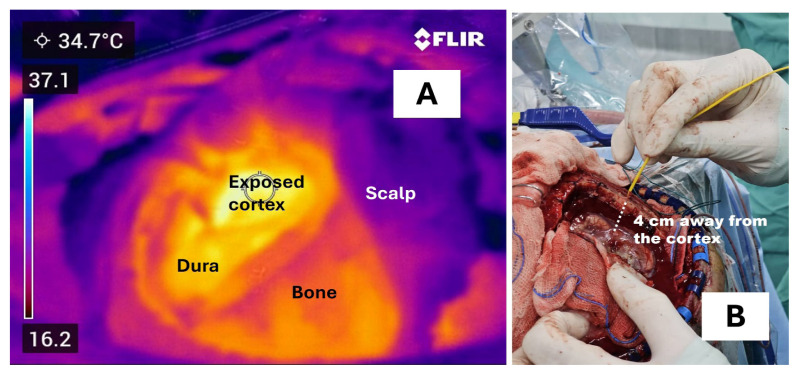
Brain and thermodynamics: (**A**) brain infrared (heat or thermal) radiation during craniotomy; (**B**) irradiated heat of the brain Note: **B** = Temperature reading reached an ambient temperature at 4 cm away from the cortex. This suggests the presence of irradiated heat or infrared brain radiation even away from the surface of the cortex.

**Figure 4:**
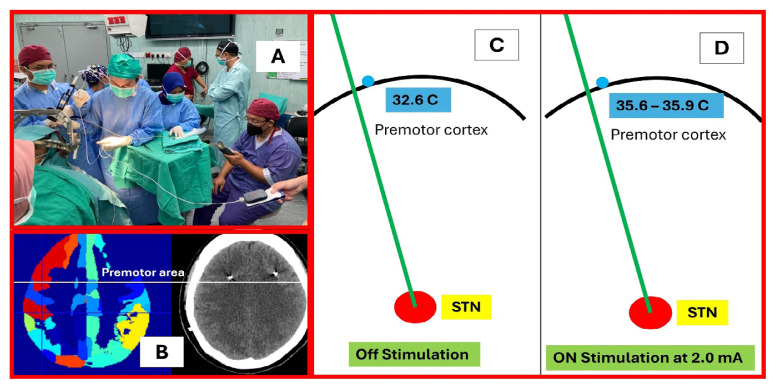
Thermodynamics and DBS: (**A**) an intraoperative view of brain temperature changes when a deep brain nucleus (STN) is stimulated; (**B**) the burr hole site for DBS surgery mapped at the premotor area; (**C**) and (**D**) an increment in cortical brain temperature noted soon after electrical currents stimulation of a deep-seated STN

With these intricate features, the brain can modulate its own temperature and enable it to have a high degree of functional freedom, perhaps through self-organisation processes such as stability-instability cycles via Hopf bifurcation or chaos brain theory (6, 7). Our ongoing study (ethical approval code: USM/JEPeM/KK/23050379) has revealed the presence of brain infrared (heat or thermal) radiation during craniotomy (Figure 3) and increased cortical brain temperature soon after electrical currents stimulation of a deep-seated subthalamic nucleus (STN) during deep brain stimulation (DBS) surgery (Figure 4). These findings may invigorate the debate on plasma-like brain dynamics (PBD) with irradiated infrared (heat) energy.

## Plasma-like Brain Dynamics

**Figure 5:**
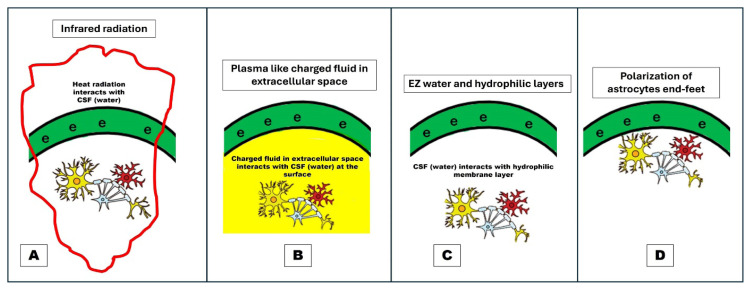
The brain’s surface current or electrons and its possible mechanisms. (A) interaction of heat with CSF; (B) interaction of plasma energy with CSF; (C) interaction of cellular membrane with water; (D) polarisation of astrocytes Notes: **A** = The interaction of invisible infrared (heat) light emitted by a black body (brain) with water (CSF) could generate cortical surface electrons; **B** = an interaction of plasma soup with CSF at the cortical surface could also generate cortical surface electrons; **C** = an interaction of the hydrophilic layer of the cellular membrane with water (Pollack exclusion zone or EZ water) may also create cortical surface electrons; **D** = a commonly described mechanism whereby the polarisation of astrocyte end-feet could generate electrons

The brain is composed of neurons, glial cells, CSF, blood vessels, charged ions, charged molecules, polar solvents such as charged water, gaseous ions (the abundant presence of CO_2_ is easily converted to gaseous ions when electricity or electron movement is applied to them), and electrons (electricity). A combination of charged ions, charged molecules, charged water, gaseous ions, and electrons in the brain’s extracellular space may form plasma soup or electrified fluid. Noteworthy, the presence of neuronal electromagnetic brainwaves or field in close contact with the possible presence of plasma soup may form magnetohydrodynamics force, which has the following peculiar features: rapid, magnetic reconnection and magneto-acoustic waves that help to spread fast and broader brain energy or information (8, 9). Based on these salient points, the brain surface (cortical) electrons which were noted to be present and were explained as arising from glial polarisation (10), can also be explained by:

- the interaction of invisible infrared (heat) light emitted by a black body (brain) with water (CSF), as noted in a recent physics study performed by Wang and Pollack (11);
- plasma soup interaction with CSF at the cortical surface (8, 9); or
- formation of CSF (water) exclusion zone with electrons or also known as Pollack exclusion zone (EZ) water when water interacts with hydrophilic layers of cellular membrane (12) (Figure 5).

## Conclusion

The cranium houses the brain, with no visible light allowed directly into it, which may create a black box. Therefore, the brain is observed as a black body with interesting physics features, including a microgravity environment (buoyancy), black-body radiation, brain thermodynamics, and the possible presence of plasma-like brain energy.
